# Treatment of Placenta Prævia

**Published:** 1886-03

**Authors:** R. L. Banta

**Affiliations:** Buffalo, N. Y.


The Buffalo Medical and Surgical Journal

Vol. XXVI. MARCH, 1886. No. 8.

### Original Communications.

### *Treatment of Placenta Prævia.

By R. L. Banta, M. D., Buffalo, N. Y.

An able article on "Combined Turning in the Treatment of Placenta Prævia," by Richard Lomer, Assistant to the University Hospital for Women, in Berlin, appeared in the December number, 1884, of the *American Journal of Obstetrics.*

This "combined turning" is the method first introduced by Braxton Hicks, and the results obtained by him, by Lomer, and by others, mentioned in this paper, are very gratifying.

Every operation in obstetrics which simplifies the rectification of an abnormal birth, and which is less dangerous to the mother at the time, or afterwards, is the operation which every honest physician seeks.

One man's experience in private practice is not much; but every little helps, and my mite is now offered you.

Previous to the year 1885, I met with five cases of placenta prævia—three in consultation, and two in my own practice.

*Read before the Buffalo Obstetrical Society, Dec. 22, 1885.

Two of these cases were followed by severe attacks of puerperal septicæmia; one had a mild attack and one phlegmasia alba dolens.

Three of the infants were born dead, and one died in about a week. Two of these women had been treated by tampons for repeated hemorrhages; then dilatation with rubber bags, passing the hand into the uterus, turning, and rapid delivery. Two in the same manner, with the exception of the tampons; one, and the last, by the aid of the forceps, the cervix being sufficiently dilatable to do so after a slight effort.

Antiseptic intra uterine injections were used immediately after delivery in all these cases but one, and that one was followed by no bad results.

During the year 1885, three cases of placenta prævia came under my charge; two of them, and one partly, were treated by the method of Braxton Hicks, or a modification of his method; and this paper is written with a view of giving an account of this method, or a modification of it, and the results obtained therefrom.

Braxton Hicks' combined method consists in complete anæsthesia of the patient; sufficient dilation of the cervix to permit the entrance of one or two fingers; the introduction of one hand into the vagina, and one or two fingers into the cervix; the other hand, placed upon the abdomen, moves the breech in one direction, while the internal hand moves the presenting part in the opposite direction.

Dr. Barnes says: "The movements by which this is effected are a combination of continous pressure and gentle impulses or taps on the head or shoulder, and a series of half sliding, half pushing impulses, with the palm of the hand outside." As soon as turning is accomplished, rupture the membranes, having passed boldly through the placenta if it is in your way, take hold of a foot, leg, or if necessary, hook a finger into the fold of the thigh, pull it down until there is pressure enough to stop the bleeding, and then let nature do the rest.

By this method you avoid the danger of infection by the use of tampons. Nature's plug is vastly superior, and experience has shown that gentle traction of the foot makes pressure on the bleeding vessels, and at once stops the hemorrhage.

Of course, if the cervix is cone-shaped, long and rigid, and the finger cannot be introduced, and there is hemorrhage, the antiseptic plug must be used, until there is sufficient dilatation to pass at least one finger. But very seldom will it be found necessary to use the tampon; for, although each succeeding hemorrhage becomes more severe, in placenta prævia, generally, the os dilates sufficiently for your purpose before there is any cause for anxiety from loss of blood.

Cervical laceration and atony of the womb are very much less to be feared; for the slightest force brought to bear on the foot or leg seems to wake the womb into activity, and, as each pain becomes stronger, the soft parts of the child gradually distend the os, and labor is terminated by Nature's own process.

The use of tampons, dilating with rubber bags or in any other way, sufficient to use the forceps or pass the hand for the purpose of turning and passing the hand into the uterus, no matter how well disinfected, has always been, to me, a matter of no trivial importance, takes up much valuable time, thereby enhancing the danger of infection and exhaustion from loss of blood. But turning early, by the plan under consideration, avoids these dangers, by the comparatively short time in which it can be done, arresting the bleeding vessels immediately, and also by the slight internal manipulations necessary to perform it.

In regard to the prognosis of the child by the use of this method, I can only refer you to Lomer's paper for this and many other valuable points. He has shown, by a collection of statistics, that the results obtained by this method are "no worse than those hitherto obtained;" and he says: "The value of the mother's life being incomparably greater than that of the child, it is not right to allow the mother to run even the risk of fatal hemorrhage, in order to save a child's problematic life."

Shortly after reading Lomer's paper, with the firm conviction that the method rècommended by him was far superior to any now known, I was called to see a primipara, about seven months advanced in pregnancy, for a slight flooding, which had ceased before my arrival.

Digital examination revealed nothing; external palpation, that it was a head presentation. No treatment was recommended but rest. In a week another hemorrhage occurred, which was repeated at short intervals, each time becoming more severe, making the diagnosis of placenta prævia a matter of little doubt. On March 15, 1885, the last and most severe hemorrhage took place, and, on making a digital examination, the os was found sufficiently open to pass a finger, and a portion of the placenta was plainly felt.

The friends were anxious that something should be done, for, in their eyes, things began to look rather dubious for the safety of the mother; and, with their cordial approval, steps were at once taken to put into execution the method of Braxton Hicks.

Sending for Dr. Walter Greene, of this city, who kindly brought with him Dr. Youngs, now of Warsaw, we placed the patient thoroughly under the influence of chloroform.

While manipulating the abdomen of the patient in a sort of preparatory way, there seemed to be such extreme fœtal mobility within the uterine cavity, that the thought struck me that version might be accomplished without the aid of the internal hand, thereby lessening the danger of rupturing the membranes, which would make the turning more difficult,—for we all know that the slightest handling of the membrances will sometimes break them,—also shortening the period of time necessary to keep the internal hand in the vagina, and giving it much less work to do when there.

The thought was hardly presented to my mind before it was put into practice, and, in about three minutes, version was easily performed by means of both hands on the outside, and, when done, one hand was introduced into the vagina, a finger into the cervix, taking hold of the foot and bringing it down, nature doing the rest in about an hour and a half. Unfortunately, in seizing the foot, the cord was also awkwardly pulled down, ending all chance of saving the child. An antiseptic vaginal injection was used, and my patient never had a bad symptom follow.

In about two months, another woman, a multipara, with about the same history as the above, only that the hemorrhages were more severe, consequently more dangerous to life from exhaustion, came under my charge. Prof. Tremaine, of this city, was called in consultation; but as there was no bleeding when he arrived, a little more delay was advised. In five days, another more severe and continuous hemorrhage occurred, and now the advisability of further delay was out of the question.

After the patient was completely under the influence of the anæsthetic (Dr. Tremaine being present), we tried, by external manipulation, to make out the position of the child, which we were quite unable to do, as the slightest touch brought on a pain strong enough to baffle our efforts in that direction; nor did a digital examination help us, although the os was dilated about the size of a silver dollar, and the placenta, more than quarter covering it, could be distinctly felt.

On the introduction of the hand into the vagina, the finger pushing the presenting portion of the placenta aside came in contact with a foot, which, after rupturing the membranes, was seized and pulled down.

Hemorrhage stopped at once, strong pains came on, and a macerated fœtus, about seven months advanced, was born in an hour. An antiseptic intra-uterine injection was used, and this patient also had the same good luck afterwards as the former.

On September 2d of this year, I was called to see a woman, a multipara, flowing profusely. She had been attended by another physician for three former attacks. On digital examination, found os sufficiently dilated to admit two fingers, the head presenting, and a recognizable portion of the placenta. There was considerable fœtal mobility, and, as her anxious friends were eager for any operation which might obviate the now too palpable danger, and as the flowing was considerable and time precious, chloroform, with the assistance of the nurse, was immediately administered, and the opportunity seized to make use of the plan which in my former case had proved so successful.

After version was performed, and it was easily done by external manipulation alone, one hand was passed into the vagina, the membranes ruptured, and a foot gently pulled down, labor terminating in about an hour and three-quarters. The mother did not have a bad symptom afterwards, and the child is now living.

The good results which followed the mothers in these three cases make me sincerely believe that Braxton Hicks' method is the best now known. "It is not a method which pretends to be unique and universal. Many cases are best treated according to it; others are best treated expectantly, or by rupture of the membranes. We do not pretend to do away with the tampon altogether, but we wish to show that its application is very often superfluous, and even dangerous."

Here, in two of our own cases, the method of Braxton Hicks' was not exactly followed, and possibly my plan could not be followed very often for want of fœtal mobility; but when it can be put into practice, it seems to me still an improvement.

Braxton Hicks had observed the success of certain German operators by the method of turning by external manipulations: he combined this method with ordinary version, thereby discovering a new method, which has taken his name.

My plan is, to turn by external manipulation alone, without the aid of the internal hand, and then follow his method in every other way.

